# Noise-Corrected, Exponentially Weighted, Diffusion-Weighted MRI (niceDWI) Improves Image Signal Uniformity in Whole-Body Imaging of Metastatic Prostate Cancer

**DOI:** 10.3389/fonc.2020.00704

**Published:** 2020-05-08

**Authors:** Matthew D. Blackledge, Nina Tunariu, Fabio Zugni, Richard Holbrey, Matthew R. Orton, Ana Ribeiro, Julie C. Hughes, Erica D. Scurr, David J. Collins, Martin O. Leach, Dow-Mu Koh

**Affiliations:** ^1^Division of Radiotherapy and Imaging, The Institute of Cancer Research, London, United Kingdom; ^2^Department of Radiology, The Royal Marsden NHS Foundation Trust, London, United Kingdom

**Keywords:** whole-body diffusion-weighted MRI, metastatic prostate cancer, apparent diffusion coefficient, imaging biomarker uncertainty, imaging biomarker reproducibility

## Abstract

**Purpose:** To characterize the voxel-wise uncertainties of Apparent Diffusion Coefficient (ADC) estimation from whole-body diffusion-weighted imaging (WBDWI). This enables the calculation of a new parametric map based on estimates of ADC and ADC uncertainty to improve WBDWI imaging standardization and interpretation: NoIse-Corrected Exponentially-weighted diffusion-weighted MRI (niceDWI).

**Methods:** Three approaches to the joint modeling of voxel-wise ADC and ADC uncertainty (σ_ADC_) are evaluated: (i) direct weighted least squares (DWLS), (ii) iterative linear-weighted least-squares (IWLS), and (iii) smoothed IWLS (SIWLS). The statistical properties of these approaches in terms of ADC/σ_ADC_ accuracy and precision is compared using Monte Carlo simulations. Our proposed post-processing methodology (niceDWI) is evaluated using an ice-water phantom, by comparing the contrast-to-noise ratio (CNR) with conventional exponentially-weighted DWI. We present the clinical feasibility of niceDWI in a pilot cohort of 16 patients with metastatic prostate cancer.

**Results:** The statistical properties of ADC and σ_ADC_ conformed closely to the theoretical predictions for DWLS, IWLS, and SIWLS fitting routines (a minor bias in parameter estimation is observed with DWLS). Ice-water phantom experiments demonstrated that a range of CNR could be generated using the niceDWI approach, and could improve CNR compared to conventional methods. We successfully implemented the niceDWI technique in our patient cohort, which visually improved the in-plane bias field compared with conventional WBDWI.

**Conclusions:** Measurement of the statistical uncertainty in ADC estimation provides a practical way to standardize WBDWI across different scanners, by providing quantitative image signals that improve its reliability. Our proposed method can overcome inter-scanner and intra-scanner WBDWI signal variations that can confound image interpretation.

## 1. Introduction

Whole-body diffusion-weighted MRI (WBDWI) is fast gaining acceptance as a powerful tool for diagnosing, staging, and assessing the response of myeloma (1), lymphoma (2), and metastatic prostate (3, 4) and breast (5) cancers to systemic treatments. WBDWI provides high contrast between disease and healthy tissue, without the need for exogenous contrast agent injection. The technique has been shown to be particularly helpful in defining the extent of metastatic bone disease, allowing quick assessment of cancer spread “at a glance.” By acquiring images with at least two diffusion weightings (*b*-values), WBDWI also enables calculation of the apparent diffusion coefficient (ADC) of tissues, a putative response imaging biomarker that reflects tumor cellularity (6); ADC increase is observed in effective treatments and early patient response to therapies (7). However, standardization of this technique is complicated due to the number of parameters that require optimization for each scanner (8).

Computed DWI (cDWI) (9) is a post-processing MRI technique that combines voxel-wise estimates of the MR signal at *b* = 0 s/mm^2^ (S0) and ADC to synthetically generate higher *b*-value images (assuming a monoexponential relationship between image signal and ADC). Synthesized *b*-values are typically higher than those that can be directly acquired on MRI scanners due to constraints in image signal-to-noise ratio (SNR) and/or measurement time; such images help to overcome the “T2 shine-through effect” that can lead to misinterpretation of metastatic disease (10), and have improved SNR compared with directly acquired high *b*-value images. By optimizing the signal from diseased tissues and minimizing the signal from normal tissues, cDWI has been applied as a pre-processing step in semi-automatic segmentation of disease in WBDWI studies, providing quantification of whole-body volumetric tumor burden (tDV) and global disease ADC (gADC) as biomarkers for treatment response (11, 12).

Estimated cDWI images are affected by the T1-relaxivity, T2-relaxivity, and proton density of the imaged tissue, such that the resulting contrast on cDWI may not wholly depend on differences in ADC. Moreover, image contrast is heavily influenced by coil sensitivity, making images susceptible to (i) signal inhomogeneities across the imaging field of view (a “bias field”), and (ii) non-uniform signal between anatomical acquisition stations on WBDWI (3). Such signal inhomogeneities hinder the development of reproducible disease segmentation techniques in WBDWI. A previous attempt to provide images with pure diffusion-weighted contrast was exponentially weighted DWI (eDWI) (13). Using eDWI, S0 is set to a constant value across the entire field of view so that the synthetic higher *b*-value images are obtained using only ADC generated contrast. However, this technique is suboptimal due to inherently low image contrast-to-noise ratio (CNR).

In this paper, we propose NoIse-Corrected, Exponentially-weighted DWI (niceDWI) as a technique to improve the limitations of previously described methods. By combining voxel-wise measurements of ADC with estimates of its statistical uncertainty, σ_ADC_ (calculated through linear weighted least-squares fitting), it is possible to apply a noise reduction weighting to conventional eDWI images and thus improve the CNR of these images. Furthermore, we hypothesize that niceDWI is less susceptible to the bias fields and inter-station signal inhomogeneities that are observed in conventional cDWI. This technique can potentially normalize the signal within and between WBMRI acquisitions, thus facilitating comparison of longitudinal WBMRI studies within and between institutions. This may lead to improved visual assessment of disease and reader confidence when using WBDWI. We also derive and assess the statistical properties of three methods for calculating σ_ADC_. The first of these, *Direct Weighted Linear Least-Squares Estimation* (DWLS), requires the acquisition of 3 or more images at each *b*-value measured (≥2 unique *b*-values), whilst the second method, *Iterative Weighted Linear Least-Squares Estimation* (IWLS), places the slightly weaker restriction that the total number of (not necessarily unique) *b*-values measured is 3 or more. Our third methodology, *Smoothed Iterative Weighted Linear Least-Squares Estimation* (SIWLS), improves precision of σ_ADC_ from IWLS in real-world WBDWI protocols where it is typical to acquire only 3 independent *b*-values (the lower bound for IWLS).

## 2. Theory

**Figure 1:**
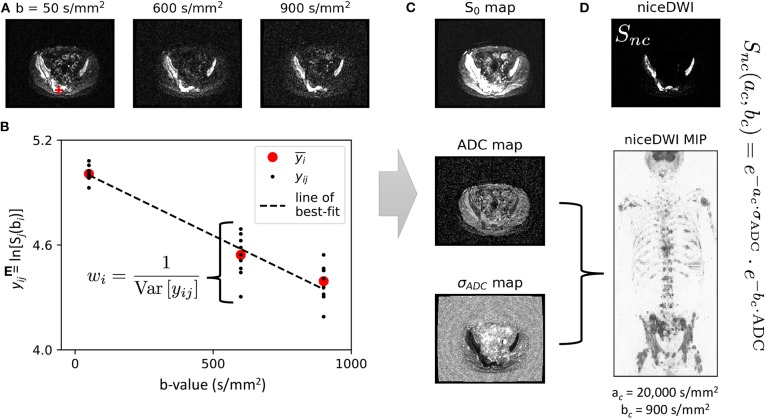
An illustration of our data workflow for niceDWI. **(A)** Axial images are acquired at three different *b*-values, with each *b*-value applied over three orthogonal diffusion-encoding gradient directions, and repeated three times for signal averaging (a total of nine image excitations per *b*-value). **(B)** For each voxel location, the nine acquisitions may be used to estimate a weight for linear regression as the inverse of the data variance each *b*-value, *w*_*i*_ = 1/Var[*y*_*ij*_]. In a conventional clinical setting, vendors would supply only the average (geometric and arithmetic mean) of these data, $\bar{y_{i}}$ (as indicated by the red circles). **(C)**. Weighted linear regression provides estimated maps of (i) signal at *b* = 0 s/mm^2^, S_0_, (ii) ADC, and (iii) ADC uncertainty σ_ADC_. Maps of ADC and σ_ADC_ may then be combined using the niceDWI signal model **(D)**, and viewed axially (top-right) and/or as a volume-rendered maximum intensity projection (MIP, bottom-right). It should be noted that we display axial diffusion weighted imaged images (*S*_*nc*_) using a grayscale colormap, where regions of low ADC and low σ_ADC_ are displayed as bright white regions against a dark background, whereas volume-rendered MIPs are displayed using an inverse grayscale where regions of low ADC and low σ_ADC_ appear dark against a white background. This is common practice in whole-body DWI applications.

The concept of niceDWI is illustrated in Figure 1. Voxel-wise estimates of ADC and the uncertainty in its estimation (σ_ADC_—characterizing the standard error of the ADC value) are calculated using weighted linear least-squares regression (WLS) to produce maps of both parameters. We combine the resultant quantitative maps to generate a novel computed image in post-processing, S_*nc*_, according to the model:

(1) $$\begin{array}{l} {\text{S}}_{nc}\left({\text{a}}_{c},{\text{b}}_{c}\right)=e^{-{\text{a}}_{c}\cdot \sigma_{\text{ADC}}}\cdot e^{-{\text{b}}_{c}\cdot \text{ADC}},\;{\text{S}}_{nc}\in \left(0,1\right) \end{array}$$

where ${\text{a}}_{c}\in {\mathbb{R}}^{+}$ and ${\text{b}}_{c}\in {\mathbb{R}}^{+}$ can be reduced/increased in real-time to provide weaker/stronger σ_ADC_ and ADC weighting in the computed image, respectively. The resulting signal intensity remains purely *quantitative* in contrast to conventional high *b*-value DWI where image intensity is influenced a multitude of factors including T1/T2-weighting, RF-receive coil sensitivity, and proton density. Images may be displayed as a volumetric 3D maximum intensity projection (MIP) that enables visualization of bone and soft tissue disease “at-a-glance,” where hot-spots reveal regions with (i) low ADC and (ii) high precision of the ADC estimate. In the remainder of this section we describe our approach to joint estimation of ADC and σ_ADC_ from noisy DWI data, and then derive models for the expected image noise in calculated σ_ADC_ maps.

### 2.1. Estimation of ADC and σ_ADC_

The conventional monoexponential model for DWI is given by

(2) $$\begin{array}{l} S\left({\text{b}}_{i}\right)=S_{0}\cdot \exp\left\{-{\text{b}}_{i}\cdot \text{ADC}\right\}+\nu_{i} \end{array}$$

where *S*_0_ represents the signal-intensity in the absence of any diffusion-weighting (*b* = 0 s/mm^2^), and $\nu_{i}\sim N\left(0,\sigma_{\nu }\right)$ is additive homoscedastic noise sampled from a univariate, zero-mean normal distribution with standard deviation σ_ν_. ADC estimation is commonly performed through linear least-squares regression (LLS) following linearization of this function by a log-transform:

(3) $$\begin{array}{l} y_{i}=\ln\left[S\left({\text{b}}_{i}\right)\right]\\ \;={\text{b}}_{i}\cdot \text{ADC}+\ln\left[S_{0}\right]+\varepsilon_{i} \end{array}$$

The magnitude of imaging noise following log-transformation becomes dependent on the acquisition *b*-value (*b*_*i*_), ADC, and *S*_0_ according to (by error propagation):

(4) $$\begin{array}{l} \varepsilon_{i}\sim N\left(0,\sigma_{i}\right),\;\sigma_{i}=\frac{\sigma_{\nu }}{S_{0}e^{-b_{i}\cdot \text{ADC}}} \end{array}$$

Such heteroscedastic noise warrants the use of a weighted linear-least squares optimization approach (WLS) for ADC estimation, as has been previously explored (14). WLS is also convenient for voxel-wise estimation of the ADC uncertainty, σ_ADC_. In this article we explore three potential strategies for WLS fitting of these parameters: Direct Weighted linear Least-Squares (DWLS), Iterative Weighted linear Least-Squares (IWLS), and smoothed IWLS (SIWLS). All algorithms were written in Python using the NumPy Einstein summation convention routines, and are provided in the Supplementary Data.

#### 2.1.1. Direct Weighted Linear Least-Squares Estimation (DWLS)

In most conventional WBDWI studies, multiple acquisitions are performed at each *b*-value, and then averaged to increase the signal-to-noise ratio (SNR) in the final trace-weighted image. Vendors typically return only these averaged images to reduce the large storage requirements for retaining individual image excitations. However, individual acquisition images provide direct calculation of weights for use in WLS estimation of ADC and σ_ADC_, as demonstrated in Figure 1. Consider that *M* repeat excitations are acquired for each of *N* different *b*-values. A weight for each *b*-value *b*_*i*_ at each pixel location can be derived as the inverse variance of the *M* data acquired at *b*_*i*_:

(5) $$\begin{array}{l} w_{i}=\frac{M-1}{\overset{M}{\underset{j=1}{\sum }}{\left(y_{ij}-\bar{y_{i}}\right)}^{2}} \end{array}$$

where $\bar{y_{i}}$ represents the average log-signal at a particular voxel location over the repeat acquisitions. DWLS proceeds by converting these weights into a diagonal matrix, **W**, and combining this with matrices representing the known *b*-values, **B**, and measured log-signals, **Y**:

(6) $$\begin{array}{l} W=\;\left(\begin{array}{ccccc} w_{11} & 0 & \cdots & \; & 0\\ 0 & \ddots & \; & \; & \;\\ \vdots & \; & w_{1M} & \; & \vdots \\ \; & \; & \; & \ddots & \;\\ 0 & \; & \cdots & \; & w_{NM} \end{array}\right),\;\text{B}=\left(\begin{array}{cc} b_{1} & 1\\ b_{1} & 1\\ \vdots & \vdots \\ b_{N} & 1\\ b_{N} & 1 \end{array}\right),\;\text{Y}=\left(\begin{array}{c} y_{11}\\ \vdots \\ y_{1M}\\ \vdots \\ y_{NM} \end{array}\right) \end{array}$$

(where *w*_*ij*_ = *w*_*ik*_). We then calculate the vector of parameter estimates $\hat{\alpha }={\left(\hat{\text{ADC}},\ln\;\hat{S_{0}}\right)}^{⊺}$, and the covariance matrix for these parameters, ${\hat{\Sigma }}_{\alpha }$, by:

(7) $$\begin{array}{l} \hat{\alpha }={\left({\text{B}}^{⊺}\text{W}\text{B}\right)}^{-1}\text{B}\text{W}\text{Y},\;{\hat{\Sigma }}_{\alpha }={\left({\text{B}}^{⊺}\text{W}\text{B}\right)}^{-1} \end{array}$$

ADC and ADC uncertainty estimation are then derived from the first element of the parameter and parameter covariance matrices, respectively:

(8) $$\begin{array}{l} \hat{\text{ADC}}=-{\hat{\alpha }}_{1},\;{\hat{\sigma }}_{\text{ADC}}=\sqrt{{\hat{\Sigma }}_{\alpha 11}} \end{array}$$

#### 2.1.2. Iterative Weighted Linear Least-Squares Estimation (IWLS)

Our second approach is an iterative solution for joint estimation of $\hat{\alpha }$ and weight matrix $\hat{W}$, with subsequent calculation of ${\hat{\Sigma }}_{\alpha }$. This method provides the advantage that weights do not need to be calculated *a-priori*, nor are repeat measurements required at each *b*-value. Using the same matrix notation outlined in Equation (6), the following algorithm is performed for each voxel at spatial location (*x, y*) within the image:

 Inputs: **B**, **Y**, *N*_*t*_, ϵ

 ${\hat{\alpha }}^{0}={\left({\text{B}}^{⊺}\text{B}\right)}^{-1}\text{B}\text{Y}$

 **for** *t* ∈ {1, 2, …*N*_*t*_} **do**

      ${\hat{W}}^{t}=\text{diag}\left(\exp\left\{2\text{B}{\hat{\alpha }}^{t-1}\right\}\right)$

      ${\hat{\alpha }}^{t}={\left({\text{B}}^{⊺}{\hat{W}}^{t}\text{B}\right)}^{-1}\text{B}{\hat{W}}^{t}\text{Y}$

      **if** $\text{max}\left(\left|{\hat{\alpha }}^{t}-{\hat{\alpha }}^{t-1}\right|\right)<\epsilon$ **then**

         **break**

      **end** **if**

 **end for**

 ${\hat{\sigma }}_{\nu }^{2}=\frac{1}{NM-2}{\left(\text{Y}-\text{B}{\hat{\alpha }}^{t}\right)}^{⊺}{\hat{W}}^{t}\left(\text{Y}-\text{B}{\hat{\alpha }}^{t}\right)$

 ${\hat{\Sigma }}_{\alpha }={\left({\text{B}}^{⊺}{\hat{W}}^{t}\text{B}\right)}^{-1}{\hat{\sigma }}_{\nu }^{2}$

 Return: ${\hat{\alpha }}^{t}$, ${\hat{\Sigma }}_{\alpha }$

where *N*_*t*_ is the maximum number of iterations, and ϵ is a convergence tolerance (in this article we set default values of *N*_*t*_ = 100 and ε = 10^−5^). The ADC and its uncertainty can be calculated from the resulting estimates of ${\hat{\alpha }}^{t}$ and ${\hat{\Sigma }}_{\alpha }$ using Equation (8) accordingly.

#### 2.1.3. Smoothed Iterative Weighted Linear Least-Squares Estimation (SIWLS)

Calculation of ${\hat{\sigma }}_{\nu }^{2}$ and ${\hat{\Sigma }}_{\alpha }$ using IWLS is only possible if *NM* ≥ 3 and *N* ≥ 2. However, for low numbers of *M* and *N*, estimates of σ_ADC_ become especially noisy (see section 2.2). We therefore propose an optional modification to the above algorithm where we replace the estimate of the weighted data variance, ${\hat{\sigma }}_{\nu }^{2}$, with a smoothed version over the entire spatial field before being used to calculate ${\hat{\Sigma }}_{\alpha }$:

(9) $$\begin{array}{l} {\hat{\sigma }}_{\nu }^{2}\left(x,y\right)\to {\hat{\sigma }}_{\nu }^{2}\left(x,y\right)⊗\rho \left(x,y,\Omega \right) \end{array}$$

where ⊗ is the convolution operation performed over spatial field (*x, y*), and ρ is some smoothing kernel with width Ω chosen by the user (with $\int_{-\infty }^{\infty }\rho \left(x,y,\Omega \right)\text{d}x\text{d}y=1$). In this article we use a box kernel, ρ(*x, y*, Ω) = rect(*x*/Ω, *y*/Ω)/Ω^2^, due to an advantage in the statistical properties of this filter, but in principle any linear or non-linear smoothing could be used to perform this additional step. Furthermore, if adequate DWI spatial resolution is acquired, smoothing may be performed over the entire 3-dimensional spatial field (*x, y, z*). Adequate choice of Ω is considered later in this article.

### 2.2. Noise Properties of σ_ADC_ Maps

Maps of ${\hat{\sigma }}_{\text{ADC}}$ generated by the methods proposed are affected by imaging noise, and it is of interest to understand the expected distribution for ${\hat{\sigma }}_{\text{ADC}}$ given true underlying values for ADC, *S*_0_ and $\sigma_{\nu }^{2}$. Under the assumption of Gaussian distributed noise for signal intensities, the estimated data variance, ${\hat{\sigma }}_{\nu }^{2}$, will be chi-square distributed:

$$\begin{array}{l} k\frac{{\hat{\sigma }}_{\nu }^{2}}{\sigma_{\nu }^{2}}\sim \chi_{k}^{2} \end{array}$$

where *k* = *MN* − 2. By making the substitution **A** = (**B**^⊺^**W**^*t*^**B**)^−1^, we conclude that the ADC uncertainty is now chi-distributed (after appropriate scaling) with *k* degrees of freedom:

(10) $$\begin{array}{l} \hat{z}=\frac{{\hat{\sigma }}_{\text{ADC}}}{\sigma_{\nu }}\sqrt{\frac{k}{{\text{A}}_{11}}}\sim \chi_{k}, \end{array}$$

where **A**_11_ (first element of matrix **A**) is a function of the true values of ADC, *S*_0_, and the acquisition *b*-values. Given the expectation and variance of a chi-distribution are $\text{E}\left(\hat{z}\right)=\sqrt{2}\cdot \frac{\Gamma \left(\left(k+1\right)/2\right)}{\Gamma \left(k/2\right)}$ and $\text{Var}\left(\hat{z}\right)=k-\text{E}{\left(\hat{z}\right)}^{2}$, respectively, and that $\text{E}\left({\hat{z}}^{2}\right)=k$ and $\text{Var}\left({\hat{z}}^{2}\right)=2k$ (${\hat{z}}^{2}$ is chi-square distributed), we have the results:

(11) $$\begin{array}{l} \text{E}\left({\hat{\sigma }}_{\text{ADC}}\right)=c\left(k\right)\sqrt{{\text{A}}_{11}}\sigma_{\nu }\text{ E}\left({\hat{\sigma }}_{\text{ADC}}^{2}\right)={\text{A}}_{11}\sigma_{\nu }^{2}\\ \text{Var}\left({\hat{\sigma }}_{\text{ADC}}\right)=\left(1-c{\left(k\right)}^{2}\right){\text{A}}_{11}\sigma_{\nu }^{2}\text{ Var}\left({\hat{\sigma }}_{\text{ADC}}^{2}\right)=\frac{2}{k}{\text{A}}_{11}^{2}\sigma_{\nu }^{4} \end{array}$$

where

$$\begin{array}{l} c\left(k\right)=\frac{\sqrt{2}\Gamma \left(\left(k+1\right)/2\right)}{\sqrt{k}\Gamma \left(k/2\right)} \end{array}$$

An important result occurs from these formulae; consider an experiment in which *M* acquisitions are acquired at *N* different *b*-values, assumed to have data variance $\sigma_{\nu }^{2}$, and weighting value **A**_11_. Then consider a second dataset for which only the average over all *M* acquisitions is provided for each of the *N* *b*-values. By the central limit theorem the data variance would be $\sigma_{\nu \left(M\right)}^{2}=\sigma_{\nu }^{2}/M$ (the number in parentheses represent the number of signal averages), which when paired with the fact that **A**_(*M*)11_ = *M*·**A**_(1)11_ results in the relationships:

(12) $$\begin{array}{l} \text{E}{\left({\hat{\sigma }}_{\text{ADC}}\right)}_{\left(M\right)}\;=\left(\frac{c\left(N-2\right)}{c\left(MN-2\right)}\right)\cdot \text{E}{\left({\hat{\sigma }}_{\text{ADC}}\right)}_{\left(1\right)}\\ \text{E}{\left({\hat{\sigma }}_{\text{ADC}}^{2}\right)}_{\left(M\right)}\;=\text{E}{\left({\hat{\sigma }}_{\text{ADC}}^{2}\right)}_{\left(1\right)}\\ \text{Var}{\left({\hat{\sigma }}_{\text{ADC}}\right)}_{\left(M\right)}=\left(\frac{1-c{\left(N-2\right)}^{2}}{1-c{\left(MN-2\right)}^{2}}\right)\cdot \text{Var}{\left({\hat{\sigma }}_{\text{ADC}}\right)}_{\left(1\right)}\\ \text{Var}{\left({\hat{\sigma }}_{\text{ADC}}^{2}\right)}_{\left(M\right)}=\left(\frac{MN-2}{N-2}\right)\cdot \text{Var}{\left({\hat{\sigma }}_{\text{ADC}}^{2}\right)}_{\left(1\right)} \end{array}$$

If we inspect the case of *N* = 3 and *M* = 9 (as per the patient the examples explored in this article), then we would expect to see an 18-fold improvement in the SNR of σ_ADC_ maps when keeping individually acquired images over using averaged data only: (1 − *c*(1)^2^)/(1 − *c*(25)^2^) = 18.36.

These equations also demonstrate that an unbiased estimator for σ_ADC_ is given by:

(13) $$\begin{array}{l} {\hat{\sigma }}_{\text{ADC}}^{\prime }=\frac{1}{c\left(k\right)}\sqrt{{\hat{\Sigma }}_{\alpha 11}} \end{array}$$

where ${\hat{\Sigma }}_{\alpha 11}$ is derived from the DWLS or IWLS algorithms, and thus the estimated variance is modified to

(14) $$\begin{array}{l} \text{Var}\left({\hat{\sigma }}_{\text{ADC}}^{\prime }\right)=\left(\frac{1}{c{\left(k\right)}^{2}}-1\right){\text{A}}_{11}\sigma_{\nu }^{2} \end{array}$$

For the SIWLS modification, it is important to consider that the effective value for *k* will change following the smoothing process, resulting in a change in the bias correction factor *c*(*k*). For the box kernel used in this article we have *k* → *k* × Ω^2^ (i.e., for a box kernel of width Ω = 3, the effective *k* would be increase by a factor of 9).

## 3. Materials and Methods

### 3.1. Monte Carlo Simulations

Monte Carlo simulations were performed to confirm the accuracy of the assumptions made in section 2 over a range of “true” SNR and ADC values (100 SNR values and 100 ADC values in the ranges 1 → 300 and 0 → 4 × 10^−3^ mm^2^/s, respectively). For each combination of SNR and ADC we sampled nine noisy signals for each *b*-value in the set *b*_*i*_ ∈ {50, 600, 900} from a Rician distribution with parameters μ = exp{−*b*_*i*_·ADC} and σ = 1/SNR (15). Using these simulated data we performed DWLS and IWLS fitting routines to obtain (unbiased) estimates $\hat{\text{ADC}}$ and ${\hat{\sigma }}_{\text{ADC}}^{\prime }$. Furthermore, we performed IWLS fitting for data consisting of the arithmetic average of the nine values simulated at each *b*-value (denoted IWLS_(9)_). Simulations were performed $N_{s}=10^{5}$ times for each combination of SNR and ADC. For each fitting method, at each SNR/ADC combination, we compared estimates $\hat{\text{ADC}}$, ${\hat{\sigma }}_{\text{ADC}}^{\prime }$, and $\text{Var}\left({\hat{\sigma }}_{\text{ADC}}^{\prime }\right)$ against simulated values for these parameters (ground truth) using the formulae:

**Table:** 

Parameter	Ground Truth	Estimated Value
ADC	ADC value used in simulation	$\bar{\text{ADC}}=\frac{1}{N_{s}}\overset{N_{s}}{\underset{i=1}{\sum }}{\hat{\text{ADC}}}_{i}$
σ_ADC_	$\sqrt{\frac{1}{N_{s}-1}\overset{N_{s}}{\underset{i=1}{\sum }}{\left({\hat{\text{ADC}}}_{i}-\bar{\text{ADC}}\right)}^{2}}$	${\bar{\sigma }}_{\text{ADC}}=\frac{1}{N_{s}}\overset{N_{s}}{\underset{i=1}{\sum }}{\hat{\sigma }}_{\text{ADC}i}$
Var(σ_ADC_)	$\sqrt{\frac{1}{N_{s}-1}\overset{N_{s}}{\underset{i=1}{\sum }}{\left({\hat{\sigma }}_{\text{ADC}i}-{\bar{\sigma }}_{\text{ADC}}\right)}^{2}}$	$\left(\frac{1}{c{\left(k\right)}^{2}}-1\right){\text{A}}_{11}\frac{1}{{\text{SNR}}^{2}}$

where **A**_11_ can be calculated directly from the known ADC values, and *k* = 25 for IWLS and *k* = 3 for IWLS_(9)_. Simulations were performed on a 3.5 GHz, 16 GB personal computer running Python.

### 3.2. Phantom Study

In order to demonstrate the contrast-no-noise (CNR) characteristics of niceDWI, we performed a phantom study using an in-house developed test-object. Our phantom consists of five cylinders containing solutions of different proportions of water, manganese chloride and sucrose, in order to generate environments with similar T2 relaxivity and ADC ranges to those observed in tumor tissues (75–1,408 ms and 0.7–1.1 × 10^−3^ mm^2^/s, respectively); cylinders were bathed in ice-water, which was then allowed to equilibrate at room temperature for ~ 1 h in order to achieve an expected temperature of 0°C within each vial (16). Diffusion-weighted images were acquired on a 1.5T system (Siemens Aera, Erlangen, Germany) with the following acquisition parameters: echo time TE = 93 ms, repetition time TR = 6 s, in-plane parallel imaging factor R = 2 (GRAPPA), image size = 128 × 128, pixel spacing 1.91 × 1.91 mm^2^, slice thickness 5 mm, pixel bandwidth 1,955 Hz/pixel, *b*-values = 50/600/900 s/mm^2^, three orthogonal diffusion encoding directions, number of signal averages NSA = 1. Imaging was repeated three times, and images acquired for each of the three orthogonal diffusion encoding directions were considered independent (under the assumption of isotropic diffusion), providing a total of nine images for each *b*-value. These data were used to calculate maps of ADC and σ_ADC_ using the IWLS algorithm, which in turn were used to generate niceDWI images over a range of a_*c*_ and b_*c*_ values: a_*c*_ ∈ (0, 1, 000, …, 50, 000) and b_*c*_ ∈ (0, 100, …, 5, 000). This experiment was repeated twice, so that the noise statistics of niceDWI could be calculated as the standard deviation of the difference in voxel intensities from both experiments within each vial for each combination of a_*c*_ and b_*c*_ (scaled by a factor of $\frac{1}{\sqrt{2}}$). From this, the CNR was calculated between cylinders 2–5 and cylinder 1 as the ratio of the difference between the average of niceDWI voxel intensities at each a_*c*_/b_*c*_ combination, and the calculated standard deviation of signal noise.

### 3.3. Patient Study

We performed whole-body niceDWI experiments in a pilot population of 16 patients with suspect metastatic bone disease from primary prostate cancer. Axial images were acquired from skull base to mid-thigh over 4-5 imaging stations, with each station consisting of 40 slices. Images were acquired on a 1.5T scanner (Siemens Aera, Erlangen, Germany) with the following acquisition parameters: echo time TE = 79 ms, repetition time TR = 12.7 s, in-plane parallel imaging factor R = 2 (GRAPPA), image size = 128 × 104 (interpolated to 256 × 208), pixel spacing 3.36 × 3.36 mm^2^ (interpolated to 1.68 × 1.68 mm^2^), slice thickness 5 mm, pixel bandwidth 1,955 Hz/pixel, *b*-values = 50/600/900 s/mm^2^, three orthogonal diffusion encoding directions, number of signal averages NSA = 1. For each imaging station, image acquisition was repeated three times such that a total of nine images were available for each *b*-value at each slice location (three orthogonal diffusion encoding directions × three repeat acquisitions; we make the assumption of isotropic diffusion for metastatic disease).

We synthesized conventionally acquired data by determining a trace weighted image for each acquisition station as the geometric mean of signal intensities over all diffusion-encoding directions, followed by the arithmetic mean of the three trace-weighted datasets acquired at each *b*-value (equivalent to NSA = 3). For these data we calculated maps of σ_ADC_ using the SIWLS algorithm using different smoothing widths Ω ∈ (0, 1, …, 50) for a box smoothing function (Equation 9). We then calculated the root-mean-square-error (RMSE) between the σ_ADC_ maps calculated using SIWLS and σ_ADC_ maps calculated using conventional IWLS when retaining all nine independent acquisitions for each *b*-value (gold standard). The RMSE was calculated for each axial image within all patient datasets, resulting in a total of 2,680 measurements from which statistics could be calculated. Furthermore, metastatic bone disease was delineated in all patients by a dedicated radiologist using an in-house developed semi-automated segmentation pipeline (11). The RMSE was also calculated within regions-of-interest for each axial image that contained disease (resulting in 1,122 measurements).

## 4. Results

### 4.1. Monte Carlo Simulations

**Figure 2:**
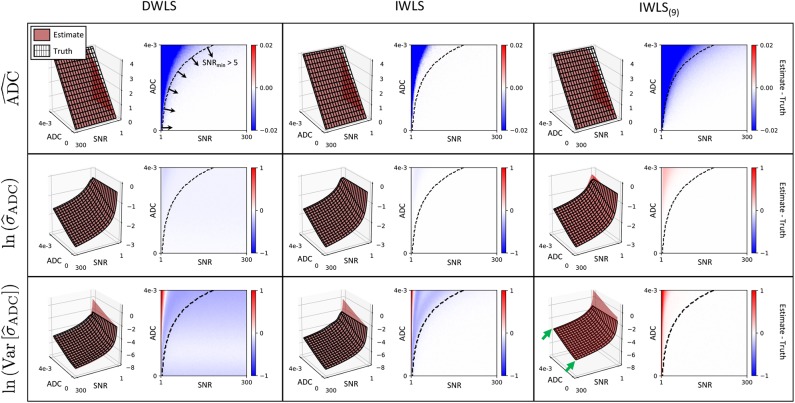
Each row demonstrates simulation results for ADC, ${\hat{\sigma }}_{\text{ADC}}$ and $\text{Var}\left({\hat{\sigma }}_{\text{ADC}}\right)$ for each of the fitting methods explored (columns). In each case, red surface plots demonstrate the estimated value derived from the equations presented in section 2, whilst the surface mesh represents the ground truth derived from simulations. The difference between estimated values and ground truth (Estimate—Truth) is also shown as a color figure on the right of each surface plot. Results for ${\hat{\sigma }}_{\text{ADC}}$ and $\text{Var}\left[{\hat{\sigma }}_{\text{ADC}}\right]$ are log-transformed for visual clarity. The IWLS scheme results in the lowest bias across all statistical estimators, especially in the region where the minimum SNR over all *b*-values is >5 (bottom-right region from dashed curve on difference images). In regions where the minimum SNR is <5, the assumption of Gaussian noise no longer holds, and results in observed differences between estimated parameters and ground truth. This is lowest for IWLS_(9)_, where averaging of data at each *b*-value improves the effective SNR. However, the statistical noise present in ${\hat{\sigma }}_{\text{ADC}}$ estimates is demonstrably poorer for IWLS_(9)_, as indicated by the generally higher $\text{Var}\left({\hat{\sigma }}_{\text{ADC}}\right)$ across all simulated ADC and SNR values (green arrows).

Figure 2 illustrates the results from the simulation study. All algorithms are able to accurately measure ADC values in the regime SNR_min_ > 5 (SNR_min_ = minimum SNR across all *b*-values) with negligible bias. Where SNR_min_ <5, ADC estimates demonstrate a negative bias, which is likely due to the effect of Rician noise in magnitude imaging data, as explored in previous studies (14) (IWLS appears to be slightly more robust to this effect). For ADC uncertainty measurements, σ_ADC_, the DWLS algorithm demonstrates a negative bias over all SNR and ADC values explored. Conversely, the IWLS and IWLS_(9)_ algorithms demonstrate no bias in the region of SNR_min_ > 5, confirming that the theoretical justifications provided in section 2 hold over these values. The same trend was observed for the noise properties of σ_ADC_, as parameterized by Var(σ_ADC_). Importantly, simulations agreed with our observation that Var(σ_ADC_) is larger when only the averaged data is available as opposed to retaining each individual acquisition at each *b*-value. Due to the bias observed in the DWLS method, we have excluded this technique from all further analysis.

### 4.2. Phantom Study

**Figure 3:**
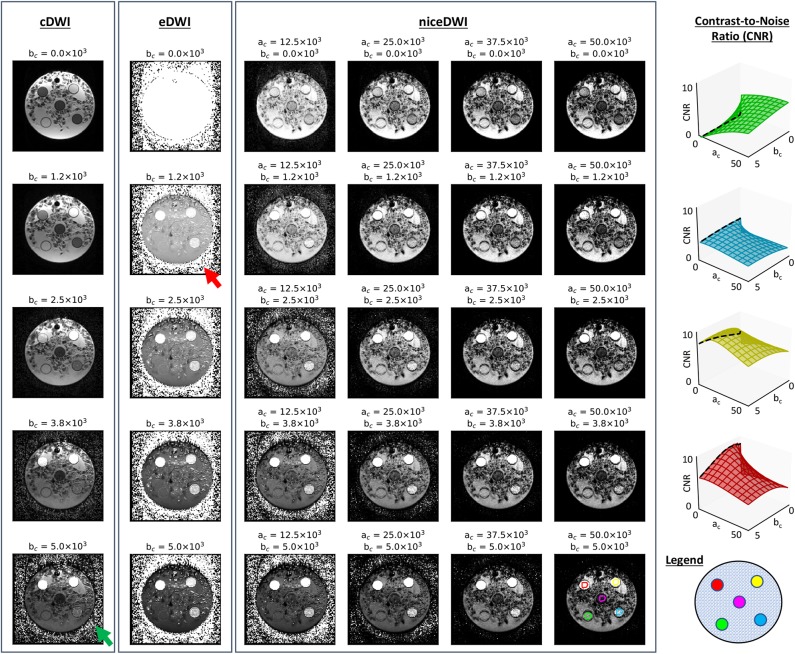
A comparison of cDWI, eDWI, and niceDWI performed for our ice-water test object over a range of computed *a*_*c*_ and *b*_*c*_ values. It is clear that a wide range of contrasts can be generated by adjusting the (*a*_*c*_, *b*_*c*_) pair in real-time, compared with cDWI and eDWI where only b_*c*_ can be adjusted. The CNR for each outer vial compared to the central vial can improved by increasing *a*_*c*_ and/or *b*_*c*_, as evidenced by the CNR surface plots illustrated on the right (color coding for these plots is presented in the bottom-right figure). It is evident that both cDWI and eDWI present with poor signal-to-background contrast (green and red arrows, respectively), and that niceDWI provides a means to alleviate this issue.

The results from our ice-water phantom study are presented in Figure 3. Our results demonstrate that contrast between the different vials can be generated in real-time by varying computed *a*_*c*_ and *b*_*c*_ values (representing increased σ_ADC_ and ADC weighting, respectively). By allowing users to adjust *a*_*c*_ and *b*_*c*_ independently, we observe it is possible to have more flexibility in the desired contrast within the image when compared to cDWI, and optimize the CNR between two regions of interest within the image. This provides the ability to increase the CNR between signal within vials and background noise compared to using eDWI alone.

### 4.3. Patient Studies

**Figure 4:**
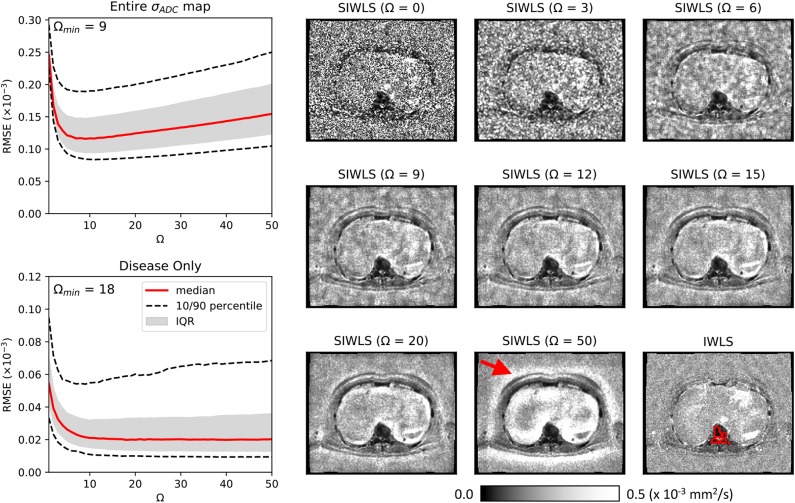
Maps of ${\hat{\sigma }}_{\text{ADC}}$ calculated using the SIWLS algorithm over different box-kernel widths Ω (right), compared to the gold-standard IWLS algorithm (bottom-right) for a single axial image from one of our patient datasets. It is evident that as the kernel width is increased, the noise field within the ${\hat{\sigma }}_{\text{ADC}}$ map is reduced and begins to more closely resemble the gold-standard map. However, as Ω is increased even further, a “haloing” effect is observed (red arrow) suggesting that an optimum Ω can be selected. On the left we present plots of RMSE calculated over the entire ${\hat{\sigma }}_{\text{ADC}}$ map, and within disease only (an example of a region of interest around disease is illustrated as the red line on the bottom-right map). Over the entire map, a minimum RMSE can be observed at Ω = 9, whilst for the disease regions only, a plateau is observed after approximately Ω = 20.

Figure 4 illustrates the results of our optimization approach for the smoothing weight Ω used in the SIWLS algorithm, along with exemplar maps of σ_ADC_ for different values of Ω in a single patient image. Results indicate that when considering the RMSE between estimates of σ_ADC_ from IWLS and SIWLS methods over the entire image, an optimum value for Ω is ~9 voxels (equivalent to 15.1 mm in this study). However, when considering voxels containing metastatic disease only, an optimum is minimum is reached once Ω>20. By inspecting the exemplar σ_ADC_ maps we observe that for Ω <20, maps appear to be affected by background “mottle” effect, which may influence the poorer RMSE within diseased regions. Conversely, as Ω reaches much higher values, σ_ADC_ maps are affected by a “halo” around the patient surface. We therefore select a value of Ω = 20 for all further exploration of the SIWLS algorithm.

**Figure 5:**
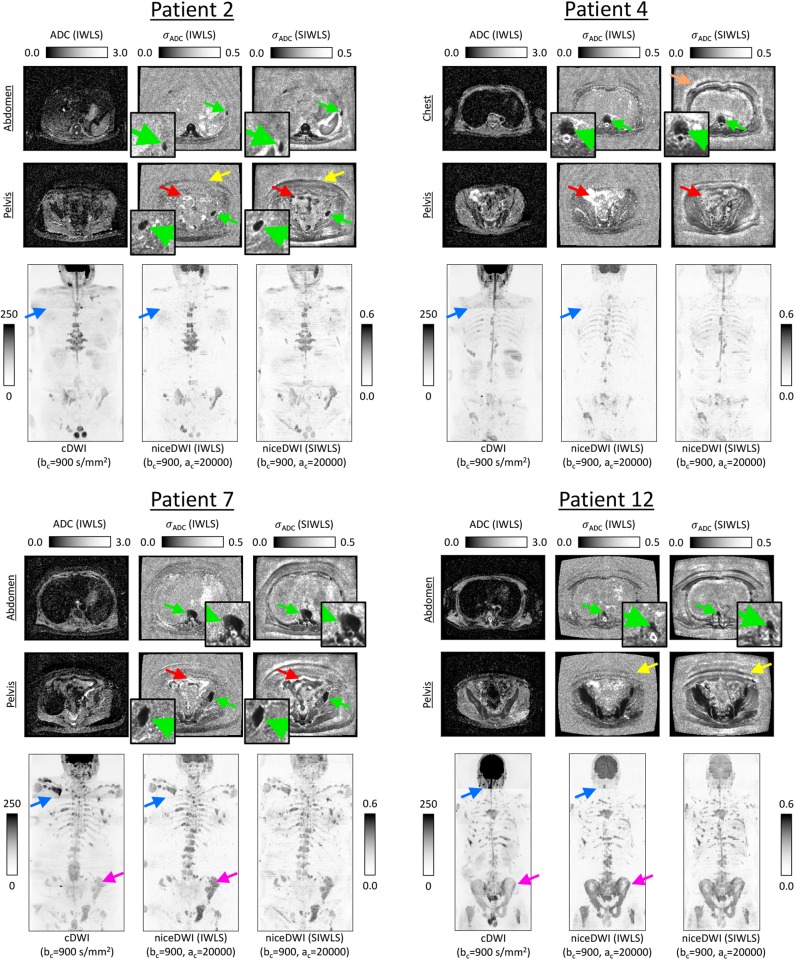
Exemplar results for four of the patients explored in this study. ADC maps for two slice locations are presented alongside σ_ADC_ maps calculated directly through the IWLS scheme, or following signal averaging with SIWLS (Ω = 20). In addition, coronal total-body MIPs of niceDWI datasets are compared alongside conventional cDWI datasets (bottom row in each case); within MIPs dark regions represent voxels with low ADC and low σ_ADC_. From the σ_ADC_ maps it is clear that the SIWLS algorithm is able to accurately measure estimates of ADC uncertainty within regions of disease (green arrows—zoomed regions also displayed) when compared with the IWLS algorithm (gold-standard). However, SIWLS is less able to recreate the appearance of σ_ADC_ in regions of peripheral fat (yellow arrows), and may be less accurate in regions where motion occurs including the bowel loop (red arrows), or close to tissue boundaries (orange arrow). By comparing the MIPs, it is shown that niceDWI can remove some of the intensity non-uniformities observed between different imaging stations on cDWI (blue arrows). Furthermore, niceDWI may be able to enhance the visualization of bone disease (purple arrows).

Figure 5 present results for four of the patient datasets, comparing total-body niceDWI images obtained from IWLS and SIWLS algorithms ($a_{c}=20,000\;{\text{s/mm}}^{2}$, $b_{c}=900\;{\text{s/mm}}^{2}$) with conventional cDWI images ($b_{c}=900\;{\text{s/mm}}^{2}$). Maximum Intensity Projections (MIPs) of these datasets illustrate that images generated with IWLS and SIWLS algorithms produce similar image contrast for evaluating tumor load in these patients. Comparing these images with MIPs from cDWI demonstrates the ability of niceDWI to provide more uniform signal intensities over the entire patient field-of-view. Furthermore, by inspecting maps of σ_ADC_ obtained by IWLS and SIWLS in these cases, it is apparent that the latter method can provide a good surrogate method where only averaged data is provided by the vendor. Regions of discrepancy between these two techniques include (i) areas of soft-tissue motion such as the bowel loop and liver, (ii) peripheral fat, and (iii) the spinal cord.

**Figure 6:**
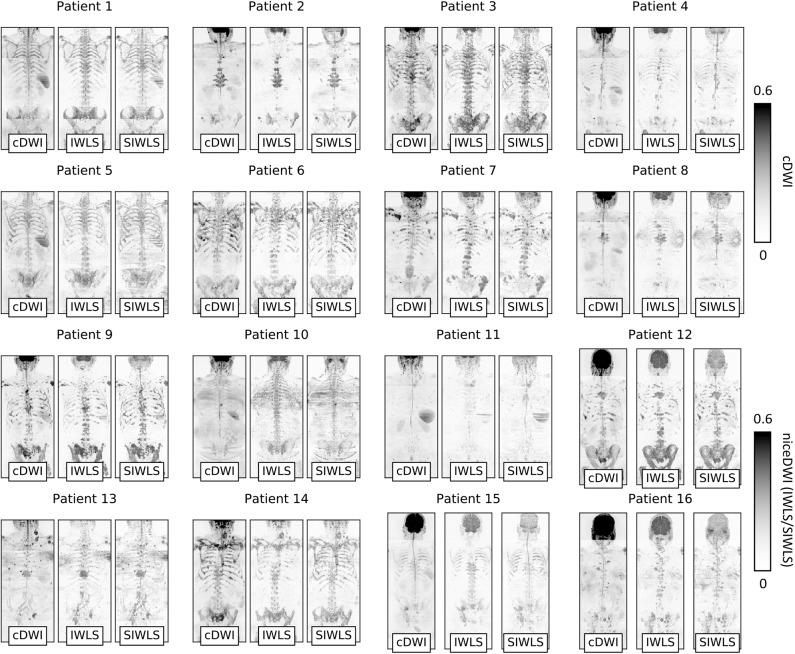
Coronal maximum intensity projections (MIPs) of cDWI and niceDWI datasets (calculated using IWLS and SIWLS) for all patients in the study. For both imaging methodologies, windowing settings have been kept constant. These examples demonstrate that niceDWI enables clear visualization of the location of regions of disease that have both (i) low ADC *and* (ii) low σ_ADC_ “at-a-glance” (dark regions represent voxels with low ADC and low σ_ADC_ and demonstrate regions of suspected disease). Furthermore, by the fact that niceDWI images demonstrate signal that is quantitative, this could provide a methodology for improving the comparison of datasets across patients by fixing windowing settings across different scans. Generally similar results are observed for both IWLS and SIWLS algorithms, with the most clear difference being the spinal cord present on SIWLS reconstructions.

An overview of niceDWI volumes (calculated using IWLS and SIWLS) for all patients is presented in Figure 6. Comparing total-body MIPs, it is evident that niceDWI provides more uniform contrast for visualization of metastatic bone disease in these patients; good agreement is observed between IWLS and SIWLS estimations methods. Some differences in contrast may be seen between cDWI and niceDWI, which is to be expected given the different properties that give rise to the derived voxel intensities, but both provide good visualization of the extent of metastatic disease.

Calculation of σ_ADC_ maps for entire WBDWI datasets using IWLS/SIWLS took ~12 s on a personal computer with a 3.5 GHz processor running Python; vast increases in speed could be expected once the algorithm has been optimized using parallel processing.

## 5. Discussion

In this article we have developed niceDWI, a post-processing approach for generating a new contrast mechanism in WBDWI studies that combines estimates of ADC with voxel-wise measurement of the uncertainty in these ADC estimates. By producing computed images in this way, we generate purely quantitative signal intensities, where bright regions (or dark on volume rendered MIPs) reflect areas of low ADC and low uncertainty in these ADC measurements. Generating purely quantitative contrast through niceDWI could offer improved visualization of treatment effects in longitudinal studies; by mitigating the effects of coil sensitivity, T1/T2-weighting and variable gain settings between scanners, this technique could enhance comparability between diffusion-weighted imaging studies acquired at different institutions. This work would be further strengthened through multi-center studies, using data acquired from multiple MR-vendors to demonstrate how well the niceDWI approach can standardize signal intensity across different patients and scanners. The diagnostic sensitivity and specificity of niceDWI could be assessed in these larger cohort studies.

We have evaluated three weighted least-squares fitting approaches (DWLS, IWLS and SIWLS) for joint estimation ADC and σ_ADC_ from WBDWI data, and established a statistical framework for exploring noise properties of σ_ADC_ maps derived from these approaches. Using this framework, we demonstrated a clear statistical advantage for retaining individual image acquisitions from WBDWI studies due to the considerable increase in the resulting SNR of calculated σ_ADC_ maps (though negligible difference is observed in ADC estimation). Although we have set up our scanners to acquire individual image acquisitions for this study, such individual acquisitions are unfortunately not conventionally retained by scanner vendors, perhaps due to data storage limitations. We have therefore also suggested a minor alteration to our IWLS algorithm to provide a potential solution when only averaged data are available (SIWLS), which could improve uptake of niceDWI in the clinical setting. As this latter methodology can be easily adapted to most clinical WBDWI protocols, it would also facilitate retrospective evaluation of niceDWI.

Through Monte Carlo simulations, we have (i) demonstrated the validity of the assumptions made in our statistical derivations, and (ii) evaluated the performance of our DWLS and IWLS model fitting approaches. Results from our study indicate that the assumptions hold in the limit of minimum SNR >5 across all *b*-values; for our patient cohort the median SNR [calculated as *S*(900)/σ_ν_] within disease voxels on *b* = 900 s/mm^2^ images was 19.8 (5th–95th percentile range: 7.4–39.8), which is well above this limit. Furthermore, it is evident that DWLS suffers from a bias in the estimation of σ_ADC_, whilst the IWLS approach remains unbiased. We therefore conclude that IWLS should be used for future studies when estimating σ_ADC_. This has the minor drawback that this approach requires multiple iterations and can thus take longer to compute, although in our patient studies we found that 97.5% of the 143 × 10^6^ voxels investigated had converged after just five iterations.

Our methodology could easily be adopted in clinical trials that utilize DWI for screening and assessing treatment response of other disease types, including in multiple myeloma (17), lymphoma, mesothelioma, and breast cancer metastasis. In future studies, repeatability measurements would be valuable to quantify what changes in niceDWI signal intensity could be considered significant following therapy; measurement of σ_ADC_ also holds promising potential for providing repeatability of ADC measurements within individual patients, although this would need to be further explored. Whilst our methodology relies on manual selection of computed *b*-values to determine the ADC weighting within niceDWI images (the choice of these parameters can be highly operator dependant), approaches such as the one developed by Gatidis et al. (18) for automatic *b*-value selection in conventional cDWI could be extended to niceDWI. Furthermore, due to low number of *b*-values typically acquired with WBDWI, we have focussed on the use of a monoexponential diffusion model in our experiments. Our approach could be extended to investigate the use of voxel-wise uncertainty estimation for parameters obtained from Intra-Voxel Incoherent Motion (IVIM) (19–21), kurtosis (22), and stretched exponential (23) diffusion imaging models.

## Data Availability Statement

The datasets generated for this study are available on request to the corresponding author.

## Ethics Statement

The studies involving human participants were reviewed and approved by Royal Marsden Hospital NHS Foundation Trust. The ethics committee waived the requirement of written informed consent for participation.

## Author Contributions

MB, NT, DC, and ML contributed the conception and design of the study. MB, FZ, JH, and ES organized the database. MB performed the statistical analysis, and wrote the first draft of the manuscript. MB and D-MK wrote the sections of the manuscript. All authors contributed to the manuscript revision, read, and approved the submitted version.

## Conflict of Interest

MB, DC, and ML are inventors on patent WO2017178227A1 relating to the published research. The remaining authors declare that the research was conducted in the absence of any commercial or financial relationships that could be construed as a potential conflict of interest. The reviewer DH declared a past co-authorship with several of the authors MB, MO, DC, ML, D-MK to the handling Editor.

## References

[1] Giles SL Messiou C Collins DJ Morgan VA Simpkin CJ West S Whole-body diffusion-weighted MR imaging for assessment of treatment response in myeloma. Radiology. (2014) 271:785–94. 10.1148/radiol.13131529 24475858

[2] Quarles Van Ufford HME Kwee TC Beek FJ Van Leeuwen MS Takahara T Fijnheer R Newly diagnosed lymphoma: Initial results with whole-body T1-weighted, STIR, and diffusion-weighted MRI compared with 18F-FDG PET/CT. Am J Roentgenol. (2011) 196:662–9. 10.2214/AJR.10.4743 21343511

[3] Koh DM Blackledge M Padhani AR Takahara T Kwee TC Leach MO. Whole-body diffusion-weighted MRI: tips, tricks, and pitfalls. Am J Roentgenol. (2012) 199:252–62. 10.2214/AJR.11.7866 22826385

[4] Eiber M Holzapfel K Ganter C Epple K Metz S Geinitz H. Whole-body MRI including diffusion-weighted imaging (DWI) for patients with recurring prostate cancer: technical feasibility and assessment of lesion conspicuity in DWI. J Magn Reson Imaging. (2011) 33:1160–70. 10.1002/jmri.22542 21509875

[5] Padhani AR Koh DM Collins DJ. Whole-body diffusion-weighted MR imaging in cancer: current status and research directions. Radiology. (2011) 261:700–18. 10.1148/radiol.11110474 22095994

[6] Koh DM Collins DJ. Diffusion-weighted MRI in the body: applications and challenges in oncology. Am J Roentgenol. (2007) 188:1622–35. 10.2214/AJR.06.1403 17515386

[7] Padhani AR Makris A Gall P Collins DJ Tunariu N De Bono JS. Therapy monitoring of skeletal metastases with whole-body diffusion MRI. J Magn Reson Imaging. (2014) 39:1049–78. 10.1002/jmri.24548 24510426

[8] Barnes A Alonzi R Blackledge M Charles-Edwards G Collins DJ Cook G. UK quantitative WB-DWI technical workgroup: consensus meeting recommendations on optimisation, quality control, processing and analysis of quantitative whole-body diffusion-weighted imaging for cancer. Br J Radiol. (2018) 91:20170577. 10.1259/bjr.20170577 29076749 PMC5966219

[9] Blackledge MD Leach MO Collins DJ Koh DM. Computed diffusion-weighted MR imaging may improve tumor detection. Radiology. (2011) 261:573–81. 10.1148/radiol.11101919 21852566

[10] Cheng L Blackledge MD Collins DJ Orton MR Jerome NP Feiweier T. T2-adjusted computed diffusion-weighted imaging: a novel method to enhance tumour visualisation. Comput Biol Med. (2016) 79:92–8. 10.1016/j.compbiomed.2016.09.022 27770679

[11] Blackledge MD Collins DJ Tunariu N Orton MR Padhani AR Leach MO. Assessment of treatment response by total tumor volume and global apparent diffusion coefficient using diffusion-weighted MRI in patients with metastatic bone disease: a feasibility study. PLoS ONE. (2014) 9:e0091779. 10.1371/journal.pone.0091779 24710083 PMC3977851

[12] Blackledge MD Rata M Tunariu N Koh DM George A Zivi A Visualizing whole-body treatment response heterogeneity using multi-parametric magnetic resonance imaging. J Algorith Comput Technol. (2016) 10:290–301. 10.1177/1748301816668024

[13] Provenzale JM Engelter ST Petrella JR Smith JS MacFall JR. Use of MR exponential diffusion-weighted images to eradicate T2 ‘shine- through' effect. Am J Roentgenol. (1999) 172:537–9. 10.2214/ajr.172.2.9930819 9930819

[14] Veraart J Sijbers J Sunaert S Leemans A Jeurissen B. Weighted linear least squares estimation of diffusion MRI parameters: strengths, limitations, and pitfalls. Neuroimage. (2013) 81:335–46. 10.1016/j.neuroimage.2013.05.028 23684865

[15] Gudbjartsson H Patz S. The rician distribution of noisy mri data. Magn Reson Med. (1995) 34:910–4. 10.1002/mrm.1910340618 8598820 PMC2254141

[16] Winfield JM Collins DJ Priest AN Quest RA Glover A Hunter S. A framework for optimization of diffusion-weighted MRI protocols for large field-of-view abdominal-pelvic imaging in multicenter studies. Med Phys. (2016) 43:95. 10.1118/1.4937789 26745903

[17] Messiou C Hillengass J Delorme S Lecouvet FE Moulopoulos LA Collins DJ. Guidelines for acquisition, interpretation, and reporting of whole-body MRI in myeloma: myeloma response assessment and diagnosis system (MY-RADS). Radiology. (2019) 291:5–13. 10.1148/radiol.2019181949 30806604

[18] Gatidis S Schmidt H Martirosian P Nikolaou K Schwenzer NF. Apparent diffusion coefficient-dependent voxelwise computed diffusion-weighted imaging: an approach for improving SNR and reducing T2shine-through effects. J Magn Reson Imaging. (2016) 43:824–32. 10.1002/jmri.25044 26348708

[19] Le Bihan D Breton E Lallemand D Grenier P Cabanis E Laval-Jeantet M. MR imaging of intravoxel incoherent motions: application to diffusion and perfusion in neurologic disorders. Radiology. (1986) 161:401–7. 10.1148/radiology.161.2.3763909 3763909

[20] Koh DM Collins DJ Orton MR. Intravoxel incoherent motion in body diffusion-weighted MRI: reality and challenges. Am J Roentgenol. (2011) 196:1351–61. 10.2214/AJR.10.5515 21606299

[21] Orton MR Collins DJ Koh DM Leach MO. Improved intravoxel incoherent motion analysis of diffusion weighted imaging by data driven Bayesian modeling. Magn Reson Med. (2014) 71:411–20. 10.1002/mrm.24649 23408505

[22] Jensen JH Helpern JA Ramani A Lu H Kaczynski K. Diffusional kurtosis imaging: the quantification of non-gaussian water diffusion by means of magnetic resonance imaging. Magn Reson Med. (2005) 53:1432–40. 10.1002/mrm.20508 15906300

[23] Bennett KM Schmainda KM Bennett RT Rowe DB Lu H Hyde JS. Characterization of continuously distributed cortical water diffusion rates with a stretched-exponential model. Magn Reson Med. (2003) 50:727–34. 10.1002/mrm.10581 14523958

