# Supplementary material for: Noise-Corrected, Exponentially Weighted, Diffusion-Weighted MRI (niceDWI) Improves Image Signal Uniformity in Whole-Body Imaging of Metastatic Prostate Cancer
Source: Front Oncol. 2020 May 8;10:704. doi: 10.3389/fonc.2020.00704 (PMC7225292; doi:10.3389/fonc.2020.00704)
Supplement: Supplementary file 1 [file Data_Sheet_1.PDF]

```python
"""
Author: Matthew D Blackledge

Copyright (c) 2020, The Institute of Cancer Research and The Royal Marsden.
All rights reserved.

Redistribution and use in source and binary forms, with or without
modification, are permitted provided that the following conditions are met:

* Redistributions of source code must retain the above copyright notice, this
list of conditions and the following disclaimer.

* Redistributions in binary form must reproduce the above copyright notice,
this list of conditions and the following disclaimer in the documentation
and/or other materials provided with the distribution.

* Neither the name of the copyright holder nor the names of its contributors
may be used to endorse or promote products derived from this software without
specific prior written permission.

THIS SOFTWARE IS PROVIDED BY THE COPYRIGHT HOLDERS AND CONTRIBUTORS "AS IS"
AND ANY EXPRESS OR IMPLIED WARRANTIES, INCLUDING, BUT NOT LIMITED TO, THE
IMPLIED WARRANTIES OF MERCHANTABILITY AND FITNESS FOR A PARTICULAR PURPOSE ARE
DISCLAIMED. IN NO EVENT SHALL THE COPYRIGHT HOLDER OR CONTRIBUTORS BE LIABLE
FOR ANY DIRECT, INDIRECT, INCIDENTAL, SPECIAL, EXEMPLARY, OR CONSEQUENTIAL
DAMAGES (INCLUDING, BUT NOT LIMITED TO, PROCUREMENT OF SUBSTITUTE GOODS OR
SERVICES; LOSS OF USE, DATA, OR PROFITS; OR BUSINESS INTERRUPTION) HOWEVER
CAUSED AND ON ANY THEORY OF LIABILITY, WHETHER IN CONTRACT, STRICT LIABILITY,
OR TORT (INCLUDING NEGLIGENCE OR OTHERWISE) ARISING IN ANY WAY OUT OF THE USE
OF THIS SOFTWARE, EVEN IF ADVISED OF THE POSSIBILITY OF SUCH DAMAGE.
"""

import numpy as np
import scipy
from scipy import ndimage
from scipy.special import loggamma

class ADCCalculation(object):
    def __init__(self, iters_max = 100, eps = 1e-5):
        """
        Parameters
        ==========
        iters_max : int
```

```python
            The maximum number of iterations for iterative weighted least-squares

    eps : float
          The tolerance at which to terminate iterative weighted least-squares estimation

    Returns
    =======
    An ADCCalcaultion instance

    """

    self.iters_max = iters_max
    self.eps = eps
    self.iters = 0

def __ck__(self, k):
    """
    (private) Calculate the bias correction factor for ADC standard deviation estimates c(k).
              Note that this is calculated using loggamma rather than gamma to avoid floating-point errors for large k.

    Parameters
    ==========
    k : float
        The total number of samples minus degrees of freedom (typically total number of samples - 2)

    Returns
    =======
    c(k) : float

    """
    k = float(k)
    return np.exp(0.5 * (np.log(2) - np.log(k)) + loggamma((k + 1) / 2) - loggamma(k / 2))

def __adc_weighting_matrix__(self, B, alpha):
    """
    (private) Calculate the weighting matrix for the iterative weighted-least squares (IWLS) ADC calculation.
              Note that this should only be used of the IWLS algorithm

    Parameters
    ==========
    B : array-like, shape (M,)
        The array of b-values used in fitting.  Repetition of b-values is allowable for multiple b-value acquisitions
```

```
        alpha : array-like, shape (N, 2)
                The current estimate of fit parameters (-ADC, log(S0)) for each pixel being calculated (assuming N pixels)

        Returns
        =======
        W : array-like, shape (N, M)
            The weighting values for each pixel N at each b-value M

        """
        B = np.c_[B, np.ones_like(B)]
        return np.exp(2 * (np.einsum("ik,jk->ij", alpha, B)))

    def data_variance(self, B, Y, alpha):
        """
        Calculate the data variance for each of the N pixels given the current parameters
        and log of the data acquired over M b-values

        Parameters
        ==========
        B : array-like, shape (M,)
            The array of M b-values used in fitting.  Repetition of b-values is allowable for multiple b-value acquisitions

        Y : array-like, shape (N, M)
            The log-data values for each pixel in N, and each b-value in M

        alpha : array-like, shape (N, 2)
                The current estimate of fit parameters (-ADC, log(S0)) for each pixel in N

        Returns
        =======
        v : array-like, shape (N,)
            The estimated data variance for each pixel in N

        """
        # B.shape = (M)
        # Y.shape = (N, M)
        # alpha.shape = (N, 2)
        M = Y.shape[1]
        W = self.__adc_weighting_matrix__(B, alpha)
        B = np.c_[B, np.ones_like(B)]
        residuals = Y - np.einsum("ik,jk->ij", alpha, B)
        return np.einsum("ij,ij,ij->i", W, residuals, residuals) / (M - 2)
```

```python
def adc_variance(self, B, alpha, v, smoothing_width=0):
    """
    Calculate the ADC parameter variance for each of the N pixels given the current parameters

    Parameters
    ==========
    B : array-like, shape (M,)
        The array of M b-values used in fitting.  Repetition of b-values is allowable for multiple b-value acquisitions

    alpha : array-like, shape (N, 2)
            The current estimate of fit parameters (-ADC, log(S0)) for each pixel in N

    v : array-like, shape (N,) or (N1, N2)
        The estimated data variance for each pixel
        If `smoothing_width' >= 1 then must be two-dimensional such that N1 x N2 = N

    smoothing_width : int (default = 0)
                      How much smoothing to apply to the data variance field, v, prior to calcaultion of the ADC variance estimator.
                      Note that if less than 1, then no smoothing is applied.
                      Number indicates the number of pixels smoothing to apply (box-car smoothing)

    Returns
    =======
    var : array-like, shape (N,)
          The estimated ADC variance for each pixel in N

    """

    if smoothing_width >= 1:
        import scipy
        smoothing_width = int(smoothing_width)
        v = ndimage.filters.uniform_filter(v, size=smoothing_width)
        v = np.clip(v, 0, None)  # For some reason, uniform spits out small negative numbers, which don't make sense.  Set to 0.

    W = self.__adc_weighting_matrix__(B, alpha)
    ws = np.einsum("ij->i", W)
    wbs = np.einsum("ij,j->i", W, B)
    wbbs = np.einsum("ij,j,j->i", W, B, B)
    adc_var = ws / (ws * wbbs - wbs * wbs) * v.ravel() # No bias correction for ADC variance
    return adc_var

def adc_std(self, B, alpha, v, unbiased=True, smoothing_width=0):
    """
```

```
        Calculate the ADC parameter standard deviation for each of the N pixels given the current parameters

        Parameters
        ==========
        B : array-like, shape (M,)
            The array of b-values used in fitting.  Repetition of b-values is allowable for multiple b-value acquisitions

        alpha : array-like, shape (N, 2)
                The current estimate of fit parameters (-ADC, log(S0)) for each pixel being calculated (assuming N pixels)

        v : array-like, shape (N,) or (N1, N2)
            The estimated data variance for each pixel
            If `smoothing_width' >= 1 then must be two-dimensional such that N1 x N2 = N

        unbiased : bool (default = True)
                   Flag to determine whether an unbiased estimate should be made

        smoothing_width : int (default = 0)
                          How much smoothing to apply to the data variance field, v, prior to calcaultion of the ADC std estimator.
                          Note that if less than 1, then no smoothing is applied.
                          Number indicates the number of pixels smoothing to apply (box-car smoothing)

        Returns
        =======
        std : array-like, shape (N,)
              The estimated ADC standard deviation for each pixel in N

        """

        c = 1.0
        if unbiased:
            if smoothing_width >= 1:
                c = self.__ck__(float(len(B) - 2) * smoothing_width * smoothing_width)
            else:
                c = self.__ck__(float(len(B) - 2))
        adc_std = (1.0 / c) * np.sqrt(self.adc_variance(B, alpha, v, smoothing_width=smoothing_width))
        return adc_std

    def weighted_least_squares(self, X, Y, W):
        """
        Perform a linear weighted least-squares optimisation on the data pair X, Y

        Parameters
```

```
    ==========
    X : array-like, shape (M,)
        The independent variables for the WLS fit

    Y : array-like, shape (N, M)
        The dependent variables for the WLS fit. The first axis represents each curve to be fit

    W : array-like, shape (N, M)
        The weighting matrix to be used.

    Returns
    =======
    alpha : array-like, shape (N, 2)
            The estimated parameters for each datum in N

    """
    ws = np.einsum("ij->i", W)
    wxys = np.einsum("ij,j,ij->i", W, X, Y)
    wxs = np.einsum("ij,j->i", W, X)
    wys = np.einsum("ij,ij->i", W, Y)
    wxxs = np.einsum("ij,j,j->i", W, X, X)
    denom = (ws * wxxs - wxs * wxs)

    a = (ws * wxys - wxs * wys) / denom
    b = (wys * wxxs - wxs * wxys) / denom
    return np.c_[a, b]

def iterative_weighted_least_squares(self, B, Y, return_iters_map=False):
    """
    Perform an interative linear weighted least-squares optimisation on the data pair B, Y.
    Note this makes the implicit assumption that ADC is being fit (as opposed to the more general WLS)

    Parameters
    ==========
    B : array-like, shape (M,)
        The independent b-values for the IWLS fit

    Y : array-like, shape (N, M)
        The dependent variables for the IWLS fit. In this case it should be the log of signal values for each pixel in N

    Returns
    =======
    alpha : array-like, shape (N, 2)
```

```python
            The estimated parameters for each datum in N as a list [-ADC, ln(S0)]

        """

        # An initial estimate
        A = self.weighted_least_squares(B, Y, np.ones_like(Y))

        # Initiaite the weights
        W = self.__adc_weighting_matrix__(B, A)

        if return_iters_map == True:
            iters_map = np.zeros(A.shape[0])

        # Loop the same operations over maximum number of iterations until convergence
        for self.iters in range(self.iters_max):
            A_ = A[:, 0]
            A = self.weighted_least_squares(B, Y, W)
            W = self.__adc_weighting_matrix__(B, A)

            mask = np.abs(A_ - A[:, 0]) > self.eps
            if return_iters_map:
                iters_map[mask] = iters_map[mask] + 1
            if np.max(mask) == 0:
                break

        if return_iters_map == True:
            result = (A, iters_map)
        else:
            result = A

        return result

    def calculate_ADC_maps(self, b_values, image_arrays, calculate_uncertainty = True, smoothing_width = 20, ADC_scale = 1e3):
        """
        Used to calculate ADC parameters from a 2D image

        Parameters
        ==========
        B : array-like, shape (M,)
            The independent b-values for the images

        image_arrays : array-like, shape (M, D1, D2)
                       List of length M, with each element being a 2D array with identical shape (D1, D2)
```

```python
    calculate_uncertainty : bool (default = True)
                            Return the ADC standard deviation estimate map

    smoothing_width : int (default = 20)
                      How much smoothing to apply to the data variance field, v, prior to calcaultion of the ADC std estimator.
                      Note that if less than 1, then no smoothing is applied.
                      Number indicates the number of pixels smoothing to apply (box-car smoothing)

    ADC_scale : What units should be applied to the resultant maps?
                Example.  If ADC_scale = 1e3 then ADC units would be 10^-3 mm^2/s

    Returns
    =======
    ADC : array-like, shape (D1, D2)
          The (scaled) ADC map

    S0 : array-like, shape (D1, D2)
         The computed b = 0 image

    ADC_std : array-like, shape (D1, D2) (optional)
              The (scaled) ADC uncetainty image

    """

    n_b_values = len(b_values)

    if not len(image_arrays) == n_b_values:
        raise Exception(ValueError, "Inputs not of identical size")

    if len(np.unique(b_values)) < 2: # Should also handle case of just 1 input b-value
        raise Exception(ValueError, "Need at least 2 distinct b_values")

    if calculate_uncertainty is True and n_b_values == 2:
        raise Exception(ValueError, "For uncertainty calcaultion, need >2 measurements")

    image_shape = np.array(image_arrays[0]).shape
    if not len(image_shape) == 2:
        raise Exception(ValueError, "Input image shapes must be 2 dimensional")
    for image in image_arrays:
        if not np.allclose(np.array(image).shape, image_shape):
            raise Exception(ValueError, "Input images are not all same shape")
```

```python
        # Create the relevant array for the signal elements
        data_shape = [np.prod(image_shape), n_b_values]
        log_data = np.zeros(data_shape)
        for i in range(n_b_values):
            log_data_ = np.log(image_arrays[i])
            log_data_[image_arrays[i] <= 0] = 0.0
            log_data[:, i] = log_data_.ravel()
        b_values = np.array(b_values)

        # Calculate the maps using these data
        A = self.iterative_weighted_least_squares(b_values, log_data)
        ADC = -1.0 * ADC_scale * A[:, 0].reshape(image_shape)
        S0 = np.exp(A[:, 1]).reshape(image_shape)

        if calculate_uncertainty is True:
            data_variance = self.data_variance(b_values, log_data, A)
            ADC_std = self.adc_std(b_values, A, data_variance, unbiased=True, smoothing_width=smoothing_width)
            ADC_std = ADC_scale * ADC_std.reshape(image_shape)
            results = (ADC, S0, ADC_std)
        else:
            results = (ADC, S0)

        return results

    def calculate_ADC_maps_volume(self, b_values, image_arrays, calculate_uncertainty = True,
                                  smoothed_uncertainty = True, smoothing_width = 20, ADC_scale = 1e3,
                                  axis = 0, callback = None):

        """
        Used to calculate ADC parameters from a 3D image

        Parameters
        ==========
        B : array-like, shape (M,)
            The independent b-values for the images

        image_arrays : array-like, shape (M, D1, D2, D3)
                       List of length M, with each element being a 3D array with identical shape (D1, D2, D3)

        calculate_uncertainty : bool (default = True)
                                Return the ADC standard deviation estimate map

        smoothing_width : int (default = 20)
```

```
                  How much smoothing to apply to the data variance field, v, prior to calcaultion of the ADC std estimator.
                  Note that if less than 1, then no smoothing is applied.
                  Number indicates the number of pixels smoothing to apply (box-car smoothing)

    ADC_scale : What units should be applied to the resultant maps?
                Example.  If ADC_scale = 1e3 then ADC units would be 10^-3 mm^2/s

    axis : int (default = 0)
           The axis over which to computed individual slices

    callback : ufunc
               A function that accepts 2 integers values: 1. the current slice index, and 2. the number of slices.

    Returns
    =======
    ADC : array-like, shape (D1, D2, D3)
          The (scaled) ADC map

    S0 : array-like, shape (D1, D2, D3)
         The computed b = 0 image

    ADC_std : array-like, shape (D1, D2) (optional)
              The (scaled) ADC uncetainty image

    """

    image_shape = np.array(image_arrays[0]).shape
    if not len(image_shape) == 3:
        raise Exception(ValueError, "Input image shapes must be 3 dimensional")
    for image in image_arrays:
        if not np.allclose(np.array(image).shape, image_shape):
            raise Exception(ValueError, "Input images are not all same shape")

    # Empty containers to hold the results
    ADC = np.zeros(image_shape)
    S0 = np.zeros(image_shape)
    if calculate_uncertainty is True:
        ADC_std = np.zeros(image_shape)

    n_slices = image_shape[axis]

    for i in range(n_slices):
        images = [image.take(indices = i, axis = axis) for image in image_arrays]
```

```python
            results = self.calculate_ADC_maps(b_values,
                                              images,
                                              calculate_uncertainty = calculate_uncertainty,
                                              smoothing_width = smoothing_width,
                                              ADC_scale = ADC_scale)
            if axis == 0:
                ADC[i, :, :] = results[0]
                S0[i, :, :] = results[1]
                if calculate_uncertainty is True:
                    ADC_std[i, :, :] = results[2]
            elif axis == 1:
                ADC[:, i, :] = results[0]
                S0[:, i, :] = results[1]
                if calculate_uncertainty is True:
                    ADC_std[:, i, :] = results[2]
            else:
                ADC[:, :, i] = results[0]
                S0[:, :, i] = results[1]
                if calculate_uncertainty is True:
                    ADC_std[:, :, i] = results[2]

            if not callback is None:
                callback(i, n_slices)

        if calculate_uncertainty is True:
            results = (ADC, S0, ADC_std)
        else:
            results = (ADC, S0)

        return results

if __name__ == "__main__":

    # Perform some tests

    # A simple function for adding rician noise to DW-images
    def random_rician(data, sigma):
        data = np.array(data) # Just in case
        return np.sqrt((data + np.random.normal(size = data.shape) * sigma)**2 + (np.random.normal(size = data.shape) * sigma)**2)

    # Genberate some random data in 3D.  3 b-values (50/600/900) with 9 acquisitions per b-value
    image_shape = (10, 100, 100)
    ADC_true = 1.0e-3
```

```python
ADC_map_true = np.ones(image_shape) * ADC_true
SNR = 6.0
b_values = np.r_[np.repeat(50., 9), np.repeat(600., 9), np.repeat(900., 9)]
dwi_images = np.array([random_rician(np.exp(-ADC_map_true*b_value), sigma = 1.0/SNR) for b_value in b_values])

# Create an ADCCalculation instance
adc_calc = ADCCalculation()

# Define a function to print progress
def update_func(idx, max_idx):
    print "%d/%d complete"%(idx, max_idx)

# Fit the parameters
ADC_est, S0_est, ADC_std_est = adc_calc.calculate_ADC_maps_volume(b_values,
                                                                  dwi_images,
                                                                  calculate_uncertainty = True,
                                                                  smoothed_uncertainty = False,
                                                                  ADC_scale = 1e3,
                                                                  axis = 0,
                                                                  callback = update_func)

print "ADC_est: %.2f (should be ~%.2f)"%(np.mean(ADC_est), ADC_true*1e3)
print "S0_est: %.2f (should be 1.00)"%np.mean(S0_est)
print "ADC_std_est: %.2f"%np.mean(ADC_std_est)
```
